# Dysphoric milk ejection reflex among Japanese mothers: a self-administered survey

**DOI:** 10.1186/s13006-024-00625-0

**Published:** 2024-03-27

**Authors:** Yukako Moriyama, Yuko Nakao, Naoko Yamamoto, Toshimichi Oki

**Affiliations:** School of Health Sciences, Faculty of Medicine, Kagoshima university, 8-35-1 Sakuragaoka, Kagoshima-shi, Kagoshima, 890-8544 Japan

**Keywords:** Dysphoric milk ejection reflex, Breastfeeding trouble, Breastfeeding support

## Abstract

**Background:**

The dysphoric milk ejection reflex (D-MER) is a reflex that causes temporary discomfort during milk ejection. D-MER develops due to the effects of hormones involved in lactation, and it has been reported that it is a physiological symptom different from postpartum depression, but the actual situation is unknown in Japan.

**Methods:**

This study was conducted using a self-administered, anonymous survey of mothers of children who had undergone health checkups at three years of age at five health centers in Kagoshima city and aimed to clarify the reality and perceptions of mothers regarding D-MER. The survey period was from May to September, 2022. The questionnaires were distributed to 389 mothers, and 216 (55.5% recovery rate) responses were received, of which 202 (valid response rate 93.5%) were included in the analysis.

**Results:**

Regarding the experience of D-MER, 202 mothers in the study population had given birth to a total of 403 children and experienced D-MER when breastfeeding 62 children (15.4%). Of the 202 mothers included in the analysis, 47 (23.3%) answered that they had experienced D-MER with at least one child while breastfeeding. Sixty-six mothers (32.7%) knew about D-MER. Compared to those who had not experienced D-MER, those who had experienced D-MER had significantly higher scores on the items related to having had trouble breastfeeding (odds ratio (OR]: 3.78; 95% confidence interval (CI]: 1.57, 9.09) and knowing about D-MER (OR 2.41; 95% CI 1.20, 4.84). Regarding symptoms, irritability (*n* = 24, 51.1%), anxiety (*n* = 22, 46.8%), and sadness (*n* = 18, 38.3%) ranked high. Coping strategies included distraction, focusing on the child, and, in some cases, cessation of breastfeeding. Thirty mothers (63.8%) answered that they did not consult anyone, citing reasons such as a belief that no one would be likely to understand their symptoms, and that they could not sufficiently explain their symptoms.

**Conclusion:**

The low level of awareness of D-MER suggests that it is necessary to inform and educate mothers and the public about the physiological symptoms of D-MER. Moreover, it is necessary to listen to the feelings of mothers with D-MER and support them in coping with their symptoms.

## Background

The dysphoric milk ejection reflex (D-MER) presents as a negative emotional reaction to the milk ejection reflex (MER), and it is said to cause a feeling of discomfort that lasts for several minutes immediately before milk secretion. This negative breastfeeding experience, called D-MER, was first confirmed by Heise in 2007 [1, 2]. Ureno and colleagues conducted an online study and found that D-MER has a prevalence of 9.1% and is characterized by sudden unpleasant symptoms such as anxiety, sadness, irritability, or panic that last less than five minutes during breastfeeding [3]. Physiological discomfort symptoms are thought to be related to the suppression of dopamine, a brain hormone and neurotransmitter that elevates and stabilizes mood [1, 2, 4]. There are many factors involved in regulating prolactin secretion, but dopamine that is secreted during breastfeeding is the main one. Dopamine suppresses prolactin secretion, and prolactin secretion increases when dopamine action decreases due to stimulation of lactation, which also promotes milk production [4–7]. Heise et al. suggested that such a decrease in dopamine during milk ejection may result in substantial or relatively short-term dopamine deficiency in mothers with D-MER [1]. A previous study found that symptoms of discomfort may be caused by dopamine deficiency [1]. Thus, D-MER develops due to the influence of hormones involved in lactation, and it has been reported that it is a physiological condition different from postpartum depression. The problem is that breastfeeding discomfort affects a woman’s desire to breastfeed, causing some mothers to breastfeed less often and wean earlier [3, 8].

Most studies of D-MER have been limited to case reports from overseas, including Europe, the United States of America, and Australia [1, 9]. In the future, we will need to clarify the physiological phenomena that differentiate those who develop D-MER from those who do not, through experimental research such as measuring hormones during breastfeeding. To minimize research that is invasive to mothers, it is necessary to collect as much information from mothers as possible through questionnaires. Only one research report regarding D-MER was found in the literature for all of Asia, a study conducted in China [10]. Previous studies in Japan have focused on anxiety and difficulty in breastfeeding; however, there are no research reports of difficulty breastfeeding due to D-MER, nor on the actual state of D-MER and the mother’s perception of it. Therefore, very little is known about D-MER, and despite the fact that some mothers experience it, it is considered to be a pathological condition with low general recognition. Thus, the purpose of this study was to clarify the actual situation of D-MER in Japan and mothers’ awareness of it, and to provide suggestions to support mothers with D-MER.

## Methods

### Study design and setting

A cross-sectional survey using a self-administered questionnaire was conducted between May and September 2022 at five of ten public health centers in Kagoshima City, Japan.

### Definition of terms

#### D-MER

D-MER is a pathological condition in which temporary unpleasant symptoms occur reflexively at the time of milk ejection [1], and unpleasant symptoms such as anxiety, sadness, irritability, and panic are experienced during breastfeeding.

#### Breastfeeding

A mother feeds her own breast milk to her baby to promote its growth.

### Sample and recruitment

#### Participants

In 2019, the number of births in Kagoshima City, the research site, was 4,771 [11]. The sample size for this study was 356 mothers with a margin of error of 5%, confidence level of 95%, and response rate of 50%. A total of 389 mothers who attended health checkups for three-year-old children at five public health centers in Kagoshima City, and who agreed to complete the questionnaire, were invited to participate in the study.

#### Recruitment procedure

We asked each of the five participating public health centers to conduct an investigation. After obtaining approval for the study, the researchers visited each health center and asked mothers who visited the center for their three-year-old child’s checkup to participate in the study. We prepared paper, pencils, and binders and asked mothers to either fill them out at the health center and drop them in the boxes provided, or to fill them out at home and mail them in. For those who chose to send the questionnaire by mail, we prepared a stamped return-addressed envelope and handed it to the subject. Some mothers may have experienced D-MER but may not have been aware of it, so we explained D-MER to them using a written explanation (Definition of D-MER). There was no reward for participation in the study.

#### Creating the questionnaire

The questionnaire items were created after discussion with a midwifery student and two faculty members specializing in midwifery, referring to previous literature from overseas [3, 4]. After the questionnaire was created, a pretest was conducted with two experts and four midwifery students, and corrections were made to parts that were difficult to understand, as well as for typographical errors and omissions.

The questionnaire consisted of five background items: (1) age; (2) educational background; (3) number of children born and their infant feeding method; (4) whether or not they had any problems with breastfeeding; and (5) who to consult when you have trouble breastfeeding. The questionnaire also consisted of two D-MER-related items regarding its recognition: (1) Do you know about D-MER?; and (2) If you know about D-MER, how did you learn about it? If you don’t know about D-MER, would you like to know more about it? Ten questions were asked of mothers who had or may have experienced D-MER after they had read the explanatory text: (1) When did the D-MER begin; (2) When you first experienced D-MER, how did you get information about it?; (3) Indicate whether you experienced D-MER with your first child, second child, third child, or other; (4) What were the symptoms of D-MER; (5) Did you take any action to deal with the symptoms of D-MER; (6) Did you consider stopping breastfeeding when symptoms of MER appeared; (7) Did you consult anyone when you experienced D-MER?; (8) If you did not consult anyone when symptoms of D-MER appeared, why not; (9) What kind of support did you receive when you consulted a medical professional; (10) What did you feel after experiencing D-MER. Questions 5), 8), 9), and 10) were answered as free response text.

### Statistical analysis

The *χ*^*2*^ test and Mann-Whitney test were used to compare the two groups of participants: those with and without D-MER, and the factors related to D-MER were investigated. Binary logistic regression analysis was then performed with the significant and near-significant items to adjust for confounding factors. Statistical analysis software SPSS version 28.0 was used for the analysis. The significance level was set at less than 5%.

In the free text field, the descriptions were coded according to meaningful content, with the aim of clarifying how to deal with the symptoms of mothers who experienced D-MER, and differences and similarities were noted for each code. The items were classified after comparison. This analysis was supervised by a qualitative research expert. In the following, category names are indicated by **[ ]**, and mothers’ narratives are indicated by “ ”.

## Results

### Questionnaire collection rate and effective response rate

Questionnaires were distributed to 389 mothers who consented to participate in the survey. Of these, 216 responses were obtained. Of the 216 participants, 14 with unclear responses, unclear symptoms, or missing data were excluded. As a result, the final number of participants for analysis was 202 (response rate: 55.5%, valid response rate: 93.5%).

### Participants’ basic attributes and D-MER related items

The participants’ ages ranged from 24 to 41 years, with an average of 34.7 ± 4.9 years as shown in Table 1. The total number of children born to the 202 mothers was 403. When asked whether they had ever had any problems breastfeeding, 132 (65.3%) answered that they had experienced “difficulties breastfeeding,” and 70 (34.7%) said they had “never had any problems breastfeeding.” Of the 132 mothers who answered that they had problems breastfeeding, 56 (42.4%) consulted their husbands or partners, 66 (50.0%) consulted family members (mother, sibling, mother-in-law, etc.), and 23 (17.4%) consulted acquaintances and friends; 96 (72.7%) had consulted a health care professional, and 5 (3.8%) mothers had not consulted anyone.

**Table 1:** Participant basic attributes and D-MER related items *n* = 202

Participants		Mean ± SD	n (%)
**Age**(years)			
	Under 30	34.7	32 (15.8)
	30–35 years old	(± 4.9)	67 (33.1)
	35–40 years old		66 (32.7)
	Over 40		37 (18.3)
**Parity**			
	Primiparous	33.3	53 (26.2)
		(± 5.1)	
	Multiparous	35.2	149 (73.8)
		(± 4.8)	
**Educational (graduation)**			
	Junior high school /high school		63 (31.2)
	Vocational school		54 (26.7)
	Junior college/university		85 (42.1)
**Number of children**			
	One	2	53 (26.2)
	Two	(± 0.8)	105 (52.0)
	Three		37 (18.3)
	Four		6 (3.0)
	Five		1 (0.5)
**Troubled experience breastfeeding**			
	experienced		132 (65.3)
	no experience		70 (34.7)
**Experience with D-MER**			
	Experienced		47 (23.3)
	No experience		155 (76.7)
**Recognition of D-MER**			
	Known		26 (12.9)
	have heard of it		40 (19.8)
	Unknown		136 (67.3)

Regarding whether they had experienced D-MER, 47 mothers (23.3%) answered that they had experienced D-MER with at least one child while breastfeeding. Of the total 403 children born to the mothers in this study, D-MER was experienced with 62 (15.4%) of them. Of 32 multiparous women (22 mothers with 2 children, 7 mothers with 3 children, 3 mothers with 4 children) who experienced D-MER, 11 (34.4%) of them (10 mothers of 2 children and 1 mother of 3 children) reported that they had experienced D-MER with all their babies. Twenty-one (65.6%) multiparous women stated that they had not experienced D-MER with any of their babies. Of the 32 multiparous mothers, 12 (37.5%) did not experience D-MER with their second or subsequent children. Forty-seven mothers provided the time of onset of D-MER when asked about the most recent experience with their child, with 12 (25.5%) indicating immediately after breastfeeding started, 18 (38.3%) indicating within 1 month after giving birth, and 17 (36.2%) indicating after 1 month postpartum. Of those who listed the specific number of months after 1 month postpartum, 1 case was after 3 months, 1 case was 1 year postpartum, and 1 case was 2 years postpartum.

Regarding mothers’ awareness of D-MER, over 60% of them said they did not know about it, 23 mothers (48.9%) who had experienced D-MER answered that they knew about D-MER in advance, and 24 mothers (51.1%) answered that they did not know. Of those mothers who answered “I don’t know” about D-MER, 101 (50.0%) said “I want to know more”, and 35 (17.3%) said “I don’t want to know”.

### Relationships between participants’ attributes and D-MER experience (table 2)

**Table 2:** Relationship between participant attributes and D-MER experience *n* = 202

	Participant(n)	D-MER	D-MER	*P* value
		experienced group (n-47) N (%)	no experience (n = 155)	
Age(years)				
	<35 (99)	29 (61.7)	70 (45.2)	0.047*^a)^
	≧ 35 (103)	18 (38.3)	85 (54.8)	
Parity				
	Primiparous (53)	15 (31.9)	38 (24.5)	0.312^a)^
	Multiparous (149)	32 (68.1)	117 (75.5)	
Educational				
(graduation)	Junior high school /high school (63)	14 (29.8)	49 (31.6)	0.796^b)^
	Vocational school (54)	15 (31.9)	39 (25.2)	
	Junior college/university (85)	18 (38.3)	67 (43.2)	
Troubled experience breastfeeding				
	Experienced (132)	40 (85.1)	92 (59.3)	0.001**^a)^
	No experience (70)	7 (14.9)	63 (40.6)	
Recognition of D-MER				
	Known(66)	23(48.9)	43 (27.7)	0.007**^a)^
	Unknown (136)	24(51.1)	112 (72.3)	

a:Chi-square test, b:Mann-Whitney U test *p < 0.05 **p < 0.01

Mothers who had experienced D-MER were significantly more likely to be younger than 35 years of age (*p* = 0.047) and more likely to have had a troubled experience breastfeeding (*p* = 0.001), and their awareness of D-MER was high (*p* = 0.007). However, there were no significant differences in childbirth experience or educational background. Refer to Table 2.

### Associated factors in binary logistic regression analysis

Binary logistic regression analysis, as shown in Table 3, was performed to consider related confounding factors, and the significant factor for having experienced D-MER was having trouble breastfeeding (odds ratio [OR] 3.77; 95% CI [confidence interval] 1.57, 9.09, *p* = 0.003), and recognition of D-MER (OR 2.40; 95% CI 1.20, 4.84, *p* = 0.014).

**Table 3:** Associated factors in logistic regression analysis

Factor	Comparison	*P* value	Odds Ration	95%CI
Age	< 35years vs ≧ 35years	0.072	1.895	0.944–3.805
Troubled experience breastfeeding	Experienced vs No experience	0.003**	3.774	1.566–9.093
Recognition of D-MER	Known vs Unknown	0.014*	2.409	1.199–4.841

binary logistic regression analysis *p<0.05 **p < 0.01

### Actual state of D-MER

Symptoms of D-MER.

Regarding symptoms, 24 (51%) of the 47 mothers who experienced D-MER reported irritability; 22 reported anxiety (47%), 18 reported sadness (38%), 15 reported restlessness (32%), 12 reported tearfulness (26%), 8 reported nervousness (17%), 3 reported impatience (6%), 2 reported homesickness (4%), and 1 reported panic (2%). Other symptoms occurred in 9 participants (19%), including loss of appetite, nausea, drowsiness, disgust, and a feeling of being robbed of all strength.

### Coping with symptoms of D-MER

Of the 47 mothers (23%) who answered that they had experienced D-MER, 13 (28%) stated that they had not done anything to cope with it. See Table 4. Twenty-eight (60%) of the mothers had implemented some kind of coping method, including **[**endure it patiently**]**, **[**distract myself**]**, **[**change or stop breastfeeding**]**, **[**seek someone who can empathize with me**]**, **[**express my emotions as they are**]**, and **[**calm myself down by directing my feelings towards the child**]**.

**Table 4:** Coping with symptoms of D-MER

Category	Code
**Endure it patiently**	・keep thinking it’s okay ・make me careless ・there are things like this, and I will endure it because the end is near ・endure while biting your lips and clenching your hands
**Distract myself**	・eat ice cream, drink coffee and take a break ・look outside, listen to music ・distract yourself by watching TV or use your smartphone ・go out and focus on something else ・distract yourself by talking to your family ・eat what you like, drink something warm, and calm down
**Change or stop breastfeeding**	・express milk and bottle feed ・mix nutrition ・stop breastfeeding and switch to artificial feeding
**Seek someone who can empathize with me**	・search the internet to find people who are in the same situation as you ・post on the net and get sympathy ・tell my husband and cry
**Express my emotions as they are**	・clear your mind by shedding a lot of tears ・stop breastfeeding once and leave and cry until you feel better ・get angry at other children’s trifles ・breastfeeding problems and tears
**Calm myself down by directing my feelings towards the child**	・seeing a child’s face heals me ・thinking about keeping my child’s nutrition and immunity, control my feelings ・make an effort to increase the enjoyment of taking lots of pictures of children

### Thoughts of mothers who stopped breastfeeding after experiencing D-MER

Of the mothers who experienced D-MER, 27 (57%) had no intention of stopping breastfeeding, 13 (28%) had considered stopping but did not, and 7 (15%) stopped breastfeeding. The following is an example of a typical comment: “I felt like a failure as a mother because I couldn’t accept my daughter naturally. I instinctively felt uncomfortable. There was nothing I could do about it. Just knowing that I had D-MER too, or that I wasn’t the only one, was a relief. I want it to be recognized by the world.”

### Presence or absence of consultation about D-MER

Seventeen mothers (36%) answered that they consulted someone when they experienced D-MER. Mothers who did not consult anyone thought that they would not be understood, that something was wrong with them, or that they could not explain themselves well enough. Of the mothers who answered that they consulted someone, 10 (59%) consulted their husband / partner, or their mother; 5 (29%) consulted family members such as siblings or a mother-in-law; 2 (12%) consulted acquaintances or friends; and 3 (18%) consulted medical staff.

### How did you feel when you experienced D-MER?

To describe their feelings when experiencing D-MER, mothers gave statements such as: “If I had known about what D-MER was earlier, I might have been able to talk to someone about it”; “After doing my own research, it’s comforting to know I’m not the only one going through this”; “I wanted to learn about it in the obstetrics and gynecology department and the mothers’ class”; “I thought I was disqualified as a mother”; and “D-MER is known by many people, and I hope for a supportive environment”.

## Discussion

In this study, we investigated D-MER in mothers who visited a health clinic for their three-year-old child’s checkup. In the mothers who breastfed, the incidence of D-MER was 15.4% (if the numerator was the number of infants with which the mother experienced D-MER). Ureno et al. investigated the incidence of D-MER in mothers who reported breastfeeding or had breastfed within the past six months and reported that the incidence was 9.1% [3]. The breastfeeding period of the target infants in this study was longer than the six-month period of Ureno et al.’s [3] study, and the onset of symptoms was one or two years after birth, depending on the infant, and this is thought to have increased the incidence of D-MER. A breastfeeding aversive response has also been recently reported [12, 13]. Some mothers may be confused by this reaction, which may have affected the numbers. Regarding the incidence rate, we believe that it is necessary to carefully confirm mothers’ awareness and conduct a prospective survey. Of multiparous women who experienced D-MER, 34% reported experiencing D-MER with all their babies, representing 5.4% of all mothers included in this study. Furthermore, 38% of multiparous women who experienced D-MER did not have symptoms after their second child. It was suggested that this could be grounds for alleviating the anticipatory anxiety of mothers who developed D-MER, fearing that they would also develop D-MER with their next child. Considering that prolactin affects dopamine, it is said that the amount of prolactin secreted is influenced by the infant’s sucking ability, the mother’s diet, and environmental toxins. A child’s sucking ability varies from child to child, and the mother’s diet and environmental toxins also vary, so we believe that this does not occur in all children [14, 15].

Of the mothers who experienced D-MER, most answered that they had trouble breastfeeding. Of the factors related to the experience of D-MER, the OR was the highest for experiencing trouble breastfeeding at 3.77, suggesting that the experience of D-MER may be related to the difficulty of breastfeeding. Dagla et al. clarified that “physical pain and discomfort associated with breastfeeding” were among the difficulties mothers had with breastfeeding [16]. In an online study, Smith reported that difficulties with breastfeeding include the infant’s poor latching ability and pain during feeding [17]. Takatsuka investigated mothers’ difficult psychological situations and showed that mothers experienced “unexpected pain” in breastfeeding and felt guilty about their child [18]. Many mothers feel pain and discomfort associated with breastfeeding. From this, it is highly possible that mothers with D-MER are hidden among mothers who complain of difficulty breastfeeding. In addition, in the previous literature, medical professionals were the most common people to consult about childcare, followed by husbands and family members [16, 19], which is consistent with the results of the present study. The husbands or partners, family members such as mother, sibling, and mother-in-law, and medical workers were consulted by mothers experiencing D-MER. This suggests the need to provide information and enlightenment regarding D-MER not only to specialists, but also to families. Dewi also reported that the focus should be on involving husbands and other family members in breastfeeding-related medical programs [20].

We found that the degree of recognition of D-MER is low, and the current situation is that many mothers are not aware of it. A significantly higher proportion of those who actually experienced D-MER answered that they knew about it, but 30 mothers of those who experienced D-MER answered that they had not consulted anyone about it. From the mothers’ stories, it can be seen that they are confused about D-MER. It is thought that D-MER is not yet generally recognized, and it is predicted that mothers experiencing D-MER are suffering from loneliness. Of those who answered that they did not know about D-MER, half of the mothers answered that they wanted to know about it. In addition, Ureno et al. reported that recognizing the presence of D-MER enabled people to understand and manage their symptoms [3]. From the above, it is considered necessary to provide mothers and the general public with information and enlightenment about, the physiological symptom – D-MER. It is expected that the psychological burden at the time of onset will be reduced, and that it will also lead to gaining a better understanding of those around them.

The most common symptoms of D-MER were irritability, anxiety, and sadness. The symptoms were similar to those reported in previous case studies [3, 9, 10]. Although there are individual differences, the occurrence of these symptoms during breastfeeding can lead to increased mental stress. As coping methods for the symptoms, the categories **[**endure it patiently**]** and **[**change or stop breastfeeding**]** were cited, suggesting that the onset of D-MER causes stress during breastfeeding. Among those who chose to **[**change or stop breastfeeding**]**, some actually stopped breastfeeding. A case report by Ureno et al. also reported that some patients chose to stop breastfeeding because of D-MER symptoms [9]. In this study, seven mothers stopped breastfeeding. It has become clear that D-MER causes women to stop breastfeeding. Categories such as **[**endure it patiently**]** and **[**change or stop breastfeeding**]** were identified as coping strategies for D-MER symptoms. This is considered to be largely due to the fact that 79% did not know about D-MER in advance, which accounted for more than half of the participants. It was thought that it was necessary to think about coping methods together and provide close support before changing the mother’s patience or breastfeeding method.

In addition, regarding mothers’ feelings when they experienced D-MER, they answered that they thought that they felt disqualified as mothers. Takatsuka clarified that mothers who choose breastfeeding are motivated by natural activities and admiration for breastfeeding [18]. It is thought that the cause of this feeling is the anguish resulting from the fact that breastfeeding, which mothers expect to be possible naturally until they experience D-MER, did not go as expected for these mothers [18]. Tawara argued that when mothers think that “a mother who cannot breastfeed is unqualified”, it is important to consider breastfeeding as an item in childcare and to emphasize that it does not determine the patient’s qualification as a mother [21]. In fact, it is said to be a necessary response for mothers when making decisions. The importance of psychological support for mothers who choose to stop breastfeeding due to D-MER was suggested.

As a coping method, some mothers cleared their minds by “expressing their true emotions”, such as through shedding tears until they were relieved. Nakata states that many mothers express their emotions to their husbands and mothers, and that it is necessary for the husbands to understand breastfeeding and for the mother’s feelings to be accepted by those around her [22]. This suggests the importance of listening to mothers experiencing D-MER. To continue breastfeeding even after developing D-MER, mothers developed their own coping strategies, such as **[**distract myself**]**, **[**seek someone who can empathize with me**]**, and **[**calm myself down by directing my feelings towards the child**]**. It became clear that mothers wanted a sense of security that they were not alone in experiencing D-MER. From the above, it was suggested that mothers with D-MER should be supported by allowing them the chance to express their feelings at the onset of symptoms, having someone to listen to their feelings, thinking about coping methods together, and supporting them closely. Frawley and McGuinness also state the need for mental health nursing support for D-MER [8].

In this study, we investigated the mothers of children who visited public health centers for health checkups for three-year-old children. By investigating not only the experiences of breastfeeding of mothers of three-year-olds, but also the experiences of breastfeeding of other children to whom the mothers had given birth, we were able to obtain many results regarding mothers’ experiences of D-MER. However, because the survey focused mainly on past breastfeeding experiences, there is a possibility that bias may have been present due to poor recall. In addition, since some of the infants were breastfeeding at the time of the survey, it is possible that some mothers who had not yet experienced D-MER may develop D-MER with their infants in the future. D-MER can also be felt during hand expressing or pumping. Because this study only investigated the experience of D-MER during breastfeeding, it is also necessary to investigate the experience of D-MER during hand expressing or pumping. Therefore, in the future, it will be necessary to limit the age of the target infants surveyed and also investigate the experience of D-MER during hand expressing or pumping so that sufficient data can be obtained.

## Conclusions

This study provided evidence that will assist in reducing anticipatory anxiety among mothers that the next child will also trigger D-MER. However, D-MER is still a commonly unrecognized condition, which may cause mothers experiencing D-MER to feel isolated. In the future, it will be necessary to provide information and education to mothers and the general public about the physiological symptoms of D-MER. People surrounding mothers need to identify mothers who are thought to be experiencing D-MER early on, listen to their feelings, think together about how to cope, and provide support.

## Data Availability

The datasets used and analyzed during the current study are available from the corresponding author on reasonable request.

## Abbreviations

D-MER: Dysphoric milk ejection reflex
MER: Milk ejection reflex
